# Comparative Electrokinetic Study of Alginate-Coated Colloidal Particles

**DOI:** 10.3390/gels9060493

**Published:** 2023-06-16

**Authors:** Viktoria Milkova

**Affiliations:** Institute of Physical Chemistry, Bulgarian Academy of Sciences, 1113 Sofia, Bulgaria; vmilkova@ipc.bas.bg

**Keywords:** alginate, cross-linking, colloidal particles, electrokinetic spectroscopy, adsorption

## Abstract

Alginates are a family of natural polysaccharides with promising potential in biomedical applications and tissue regeneration. The design of versatile alginate-based structures or hydrogels and their stability and functionality depend on the polymer’s physicochemical characteristics. The main features of alginate chains that determine their bioactive properties are the molar ratio of mannuronic and glucuronic residues (M/G ratio) and their distribution along the polymer chain (MM-, GG-, and MG blocks). The present study is focused on investigating the influence of the physicochemical characteristics of alginate (sodium salt) on the electrical properties and stability of the dispersion of polymer-coated colloidal particles. Ultrapure and well-characterized biomedical-grade alginate samples were used in the investigation. The dynamics of counterion charge near the vicinity of adsorbed polyion is studied via electrokinetic spectroscopy. The results show that the experimental values of the frequency of relaxation of the electro-optical effect are higher compared to the theoretical ones. Therefore, it was supposed that polarization of the condensed Na^+^ counterions occurs at specific distances according to the molecular structure (G-, M-, or MG-blocks). In the presence of Ca^2+^, the electro-optical behavior of the particles with adsorbed alginate molecules almost does not depend on the polymer characteristics but was affected by the presence of divalent ions in the polymer layer.

## 1. Introduction

Alginate (ALG) is a natural polysaccharide extracted from marine algae. It is a water-soluble linear block copolymer constituted by (1→4)-linked poly–L– α guluronate (G) and poly–D– β mannuronate (M) residues. The structure of the molecule can be described as a random or block-wise pattern of homo-polymeric regions of G-residues (G-block) and M-residues (M-block) divided by regions in which the two residues are in alternating sequence (MG-blocks) [1]. Therefore, the molar ratio of the residues (M/G ratio) and molecular weight, M_w_, are the main properties that determine the physicochemical and bioactive properties of alginates. The presence of carboxylic groups of residues governs the polyelectrolyte behavior of the molecule, rendering the alginate molecule as a polyanion.

Alginate meets all the advantages for usage in pharmaceutical and food applications: biodegradable, biocompatible, and mucoadhesive. Moreover, the polymer has very low toxicity, comparatively low cost, and good hydrogel-forming ability due to the interaction with divalent cations. Therefore, alginates have been extensively used for different biotechnological applications: dental impression, formulations for preventing gastric reflux, and bone tissue engineering or hydrogels for cell immobilization, transplantation, cell therapy, cell delivery, and protein, blood vessels, and drug delivery [2,3,4,5].

The remarkable feature of alginate is that, in the presence of divalent cations (Ca^2+^, Ba^2+^, Sr^2+^_,_ Mg^2+^), the polymer has gelling capability and good hydrogel-forming ability. The hydrogel can be synthesized via different cross-linking methods and produce structures that are similar to extracellular matrices of living tissues. That is why the polymer possesses great potential in biomedical applications and tissue regeneration [4,5,6,7,8].

The experimental studies on polysaccharides have shown a selective binding ability toward divalent cations and the affinity strongly depends on the composition [9,10,11]. Smidsrød [12] has shown that the affinity to divalent cations increases with the moiety of G-blocks in the molecules and the relation of the strength of binding depends on the type of cation (Sr^2+^ > Mg^2+^ > Ca^2+^ > Ba^2+^). A cross-linking Ca^2+^ ion is frequently used for the formation of alginate gels. Therefore, Ca^2+^ cations can be applied for investigations of the gelation properties, mechanism of formation, and morphological characteristics of produced gels.

The alginate gel can be depicted as a continuous polymer network swollen with water where physical cross-linked divalent ions (ion-induced junction zones) hold together a few polysaccharide chains. The ionic gelation of alginate in the presence of divalent cations was described in the framework of the ‘egg-box model’ [13,14,15]. According to the model, each cross-linking cation interacts with two adjacent G residues with two adjacent G residues from another chain, inducing the formation of a junction zone. The theory for the mechanism of gel formation was extended by considering the molecular orientation, dimer‒dimer interactions, and stoichiometry ratio between polymer molecules and divalent cations [16,17]. Moreover, theoretical approaches were developed to explore the specific interactions between alginates and non-binding ions and comparison with the behavior of typical polyelectrolytes [18].

Alginate is a highly charged polyelectrolyte. Numerous theoretical and experimental studies have been undertaken that are concerned with binding counterions to molecules [19,20]. In the framework of the concept of counterion condensation, the counterions are described as being delocalized in the volume in the polyion vicinity because of the long-range electrostatic interactions [21]. The estimation of the fraction of bounded or free monovalent counterions on the molecule was described according to the theory. However, the association of divalent counterions to the polyion is due to the formation of chemical bonds rather than through condensation since the binding occurs at a polyelectrolyte charge density below the critical value [22,23,24].

The investigation of the counterion mobility and electrical properties of alginate molecules in the solution or adsorbed on the oppositely charged surface is important because of the design of stable alginate-based formulations. Moreover, the application of alginate-based structures is governed by their stability or polymer properties [25]. The study presents the influence of the physicochemical characteristics of alginates on the electrical polarizability and stability of the model colloidal particles with an adsorbed polymer. This study provides an opportunity to distinguish the alginates and their more specific properties due to their structural characteristics (M/G ratio and molecular weight M_w_). The model particles of β-FeOOH were used in the investigation because their properties are very detailed and characterized in our previous studies [26,27,28,29].

## 2. Results and Discussion

### Characterization of Alginate Monolayer on β-FeOOH Colloidal Particles

**Figure 1:**
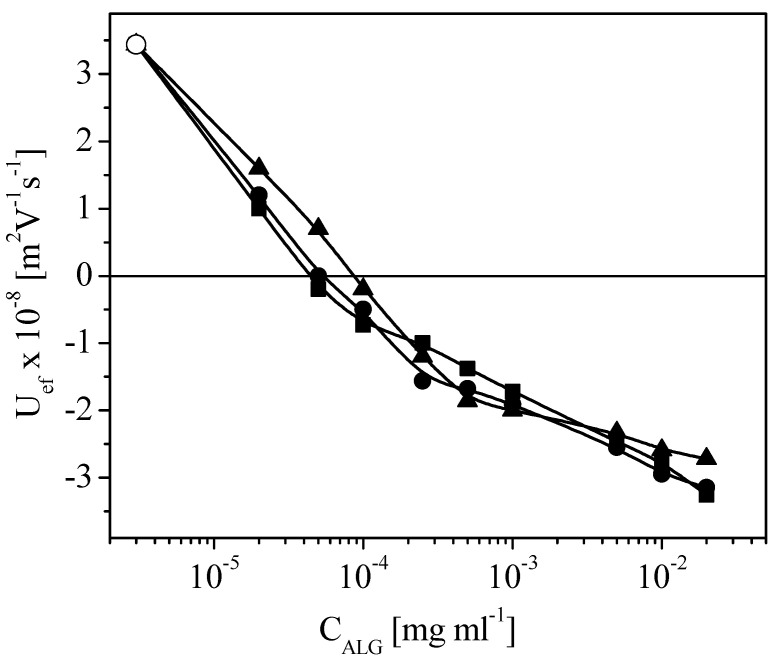
Electrophoretic mobility, U_ef_, of particles in the presence of different concentrations of alginates: A082 (▲), A122 (■), and A047 (●). Open symbol corresponding to the mobility of bare oxide particles.

The bare oxide β-FeOOH particles are positively charged (U_ef_ is ca. 3.4 × 10^−8^ m^2^ V^−1^ s^−1^) but the electrophoretic mobility became less positive as a function of the alginate concentration added to the dispersion. Figure 1 presents the electrokinetic behavior of the particles in the presence of different concentrations of alginates. Three samples of alginates with different physicochemical characteristics are used in the stability experiments: A082 (M_w_ 671.80 kDa, M/G ratio 0.82), A122 (M_w_ 687.30 kDa, M/G ratio 1.22), and A047 (M_w_ 496.70 kDa, M/G ratio 0.47).

The isoelectric point (IEP) of the particles with an adsorbed polymer is achieved in the range of ca. 3 × 10^−5^ to 10^−4^ mg/mL of alginate. The re-stabilization of the system is achieved at a higher concentration of polymer (ca. 10^−2^ mg/mL). However, despite the registered slight difference in the IEP of the particles in the presence of different alginate samples, there was no significant correlation between the particles’ electrokinetic behavior and the polymer’s characteristics.

**Figure 2:**
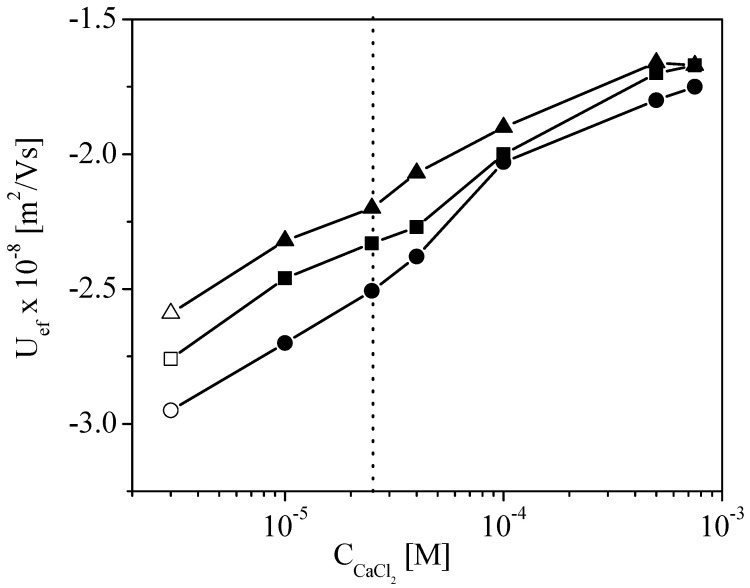
Electrophoretic mobility, U_ef_, of particles with adsorbed alginate layer in the presence of CaCl_2_: A082 (▲), A122 (■), and A047 (●). Open symbols correspond to the particles with adsorbed alginate layer without addition of CaCl_2_. The concentration of alginate in suspension is 10^−2^ mg/mL. The vertical dot line indicates the interval of CaCl_2_ concentrations with almost constant ionic strength of the suspensions.

Another possibility for a comparative investigation of the electrokinetic properties of a suspension of particles stabilized with alginates with different M_w_ and M/R ratios is based on their ability for specific interaction with Ca^2+^ ions. Figure 2 presented the electrokinetic response from stabilized suspensions (alginate concentration is 10^−2^ mg/mL) in addition to CaCl_2_. The dependencies indicate that the decreasing U_ef_ of the particles are a function of the Ca^2+^ concentration. According to the presented results, the decrease in the U_ef_ is pronounced for alginates with lower M/R ratios (if the polymers have very close M_w_, samples A082 and A122) or higher M_w_ (if the polymer M/R ratios are relative, samples A082 and A047).

The presented electrophoretic investigations are proof of a successful adsorption process. Moreover, the variation in the particles’ electrokinetic charge indicates the stability of suspension in the presence of oppositely charged polyelectrolytes or effective interactions with Ca^2+^. However, more than these results are needed to distinguish the influence of the physicochemical characteristics of alginates on the electrical properties and stability of the dispersion of particles covered with a polymer layer. The electro-optic method was applied to obtain more detailed information for the comparative investigation of the alginate samples.

The investigation of the electro-optical behavior of the suspension of non-spherical particles in the presence or absence of an adsorbed polyelectrolyte layer is a useful approach to obtain information about the stability of the system and dynamic of the counterion charge near the particle or polyion vicinity.

**Figure 3:**
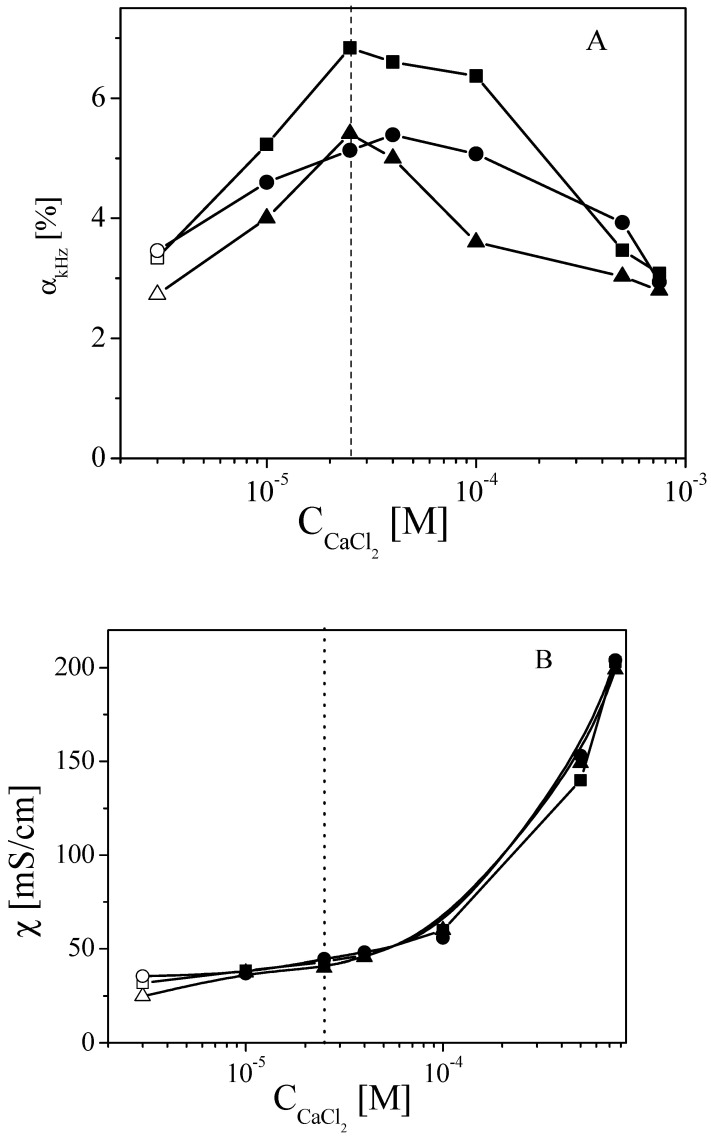
Dependence of the registered electro-optical effect, α_kHz_, (**A**) and electrical conductivity, χ, (**B**) from a suspension of particles with adsorbed alginate layer in the presence of CaCl_2_: A082 (▲), A122 (■), and A047 (●). Open symbols correspond to the particles with adsorbed alginate layer without salt. The concentration of alginate in suspension is 10^−2^ mg/mL. The vertical dot line indicates the interval of CaCl_2_ concentrations where the ionic strength of the suspensions is almost constant. The frequency of the electric field is 1 kHz. The electric field strength is 2.3 × 10^4^ V/m.

When the sinusoidal electric field is applied to the suspension of colloidal particles, two effects can generally be distinguished in the electro-optical response [30]. The first electro-optical effect can be observed at low frequencies of the applied field (below 1 KHz) if the particles possess a permanent dipole moment. The second effect (in the kHz range) is due to the polarization of the ions from the diffuse part of the double electric layer (EDL) of the particles. The mobility of these ions is equal to that of the free ions in a polyelectrolyte solution. In the suspension of particles with adsorbed fully charged polyelectrolyte, an additional effect that results from the polarization of ions with lower mobilities (condensed counterions) is observed. Figure 3 presents the variation in the kilohertz electro-optical effect, α _kHz_, from a suspension of particles with an adsorbed alginate layer in the presence of different concentrations of CaCl_2_. The experimental results indicate that the effect increases with the increasing concentration of CaCl_2_ from up to 3 × 10^−5^ M and decreases at higher salt concentrations. These observations are in line with previously reported studies on the behavior of alginate and pectin in the presence of divalent cations [29,31].

According to the electro-optic theory, when a direct current (*ν* = 0 Hz) is applied to the suspension with no spherical particles (with anisotropic electrical polarizability), all the charges of the particles are polarized (ions in the Stern layer, ions from the diffuse part of the EDL, and any associated ions). When an alternating electric field (*ν* > 0 Hz) is applied to the non-spherical particles, the particle’s orientation follows the field. Therefore, the particle rotation is observed at a low frequency of the field (*ν* < 1 kHz). With the increase in the frequency (or decrease in the period of the field) the particle is not able to follow the field and a plateau region in the frequency dependency is achieved. The estimated effect is due to the polarization of the diffuse ions from the EDL of the particles [32].

In accordance with the theory, the effect from the suspension of the ellipsoidal particles resulting from the polarization of free counterions along the long particle axis and critical frequency of relaxation *υ_cr_* can be estimated by using [33]:

(1) $$v_{cr}=\frac{{4D}_{i}^{0}}{{\pi a}^{2}}$$

where $D_{i}^{0}$ is the translation diffusion coefficient of the free ion in solution, and *a* is long particle axis. The estimated value for the particles used in the present study is very close to the experimental one (*ν_cr_*~30 kHz, $D_{Cl}^{0}$ ~2 × 10^−5^ cm^2^ s^−1^) [34] (the critical frequency of relaxation *ν_cr_* is defined as the frequency of the applied electric field corresponding to the two-fold decreases in the electro-optical effect).

**Figure 4:**
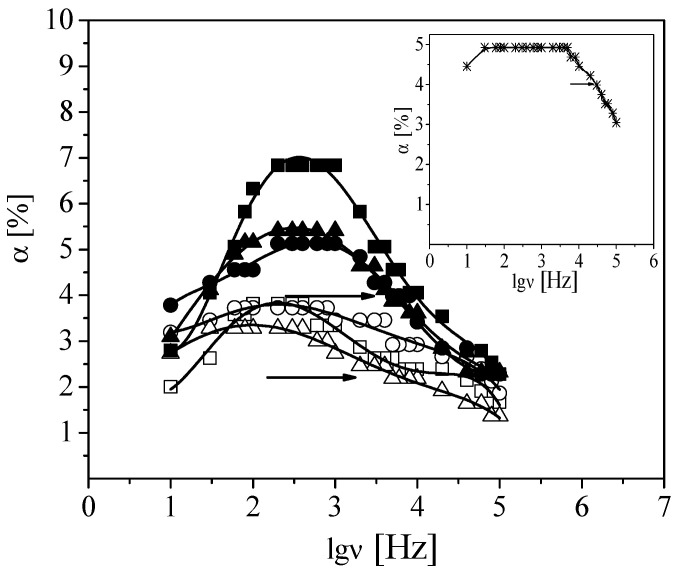
Frequency dependence of the electro-optical effect, α, from a stabilized suspension of particles with adsorbed alginate layer in the presence of 2.5 × 10^−5^ M CaCl_2_: A082 (▲), A122 (■), and A047 (●). Open symbols correspond to the particles with adsorbed alginate layer without salt. The concentration of alginate in suspension is 10^−2^ mg/mL. The electric field strength is 2.3 × 10^4^ V/m. Inset: Frequency dependence of suspension of bare particles.

As can be seen in Figure 4, the kilohertz electro-optical effect (at low energy of orientation) from suspensions of particles are covered with alginates in the absence of salt. The results indicate that the experimental value of the relaxation frequency of the effect for the polymer-coated particles is lower compared to the bare particles—*ν_cr_* (A082) ~5 kHz, *ν_cr_* (A122) ~3 kHz, and *ν_cr_* (A047) ~17 kHz. Previously, one possible explanation was shown for the similar decrease in *ν_cr_* observed for particles covered by polymers: this is related to the influence of the charge density and contour length (or molecular weight) of the polymer chain [28,29].

It is known that the polyelectrolyte behavior of ALG molecules is governed by the presence of carboxylic groups of the mannuronic (pKa~3.38) and glucuronic residues (pKa~3.65), which renders the alginate molecule as a polyanion in the presence of inorganic acids [35]. Therefore, the ALG molecule is regarded as an almost fully charged anionic polyelectrolyte during the present experimental conditions.

The classical counterion condensation theory theoretically describes the interaction of monovalent counterions with the polyion with high charge density [36,37]. Looking at the molecular structure of ALG and considering lengths of 0.435 nm and 0.517 nm for the residues of guluronic acid and mannuronic acid, respectively, can conclude that, in both cases, classical counterion condensation theory describes the interactions of counterions with the polyion with high charge density (modeled as a homogeneous linear distribution of charges) (*ξ* is 1.64, 1.38 or 1.5 in G-block, M-block, or MG-block). The calculation of *ξ* for each homo-polymeric domain does not contradict the general assumption for the condensation theory [37,38].

The theoretical critical relaxation frequency of the effect can be estimated according to the equation:

(2) $$\nu_{cr}=\frac{4D_{i}^{c}}{\pi L^{2}}$$

where $D_{Na}^{c}$ is the translational diffusion coefficient of the condensed Na^+^ counterion, $D_{Na}^{c}$ = 9.8 × 10^−7^ cm^2^ s^−1^ [34], and *L* is the polyion contour length. The length of each alginate sample is determined from the molecular weight, M/G ratio, and the length of glucuronic acid and mannuronic acid residues. The calculation indicates that the experimental values of the frequency of relaxation are higher than the theoretical ones. Therefore, we supposed that the polarization of condensed Na^+^ counterions occurs on the smaller distance, L*, (for example G-, M-, or MG-block) along the polyion contour (Table 1).

The expectation to observe different electro-optical behaviors of the particles covered by alginates with different M_w_ and M/G ratios is based on a theoretical prediction that highly charged polyelectrolyte molecules may retain part of their condensed counterions when they adsorb onto a weakly charged surface [38,39]. According to the Manning approximation, the value of the fraction *β* of free counterions near the polyion vicinity can be calculated (Table 1).

**Figure 5:**
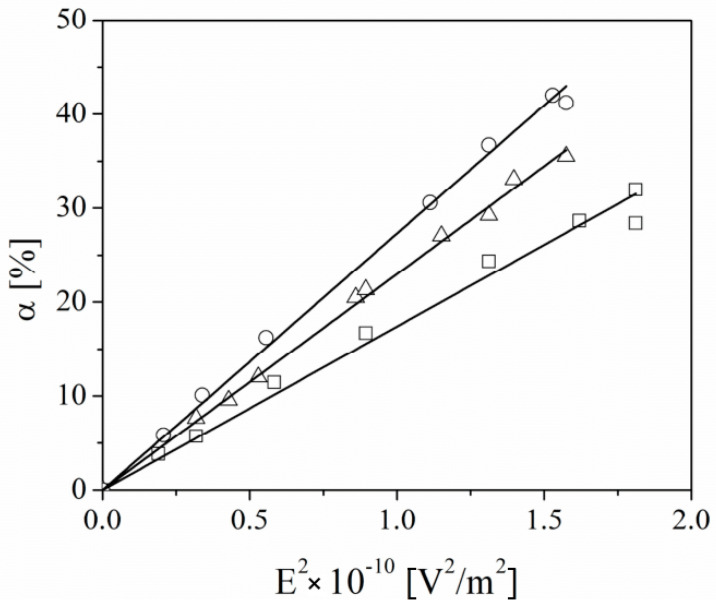
Electro-optical effect from suspensions of particles stabilized by adsorbed alginate molecules: A082 (△), A122 (□), and A047 (○). The concentration of alginate in suspension is 10^−2^ mg/mL. The frequency of the electric field is 3 kHz.

In Figure 5, the dependence of the electro-optical effect for the particles with an adsorbed alginate layer (in the absence of Ca^2+^) as a function of the electric field strength at low energy of orientation is presented. The alginate samples A082 and A122 have very closed molecular weights (ca. 680 kDa), whereas the M_w_ of sample A047 is lower. The registered kilohertz electro-optical effects of all the samples at very low energy of orientation are almost the same (ca. 3%, Figure 4). At a higher energy of orientation, the increase in the electro-optical effect correlates with the increase in a moiety of the glucuronic residues (or decrease in the M/G ratio) and decreasing in the molecular weight of the adsorbed polymers.

Figure 4 also presents the variation in the electro-optic behavior of the stabilized suspension of alginate-coated particles with CaCl_2_. The comparison between the electro-optic effect is performed at a very low salt concentration (2.5 × 10^−5^ M CaCl_2_), where the ionic strength of the suspensions is almost not affected by the presence of salt (Figure 3b). It is seen that the kHz effect from the suspension increases with the addition of Ca^2+^ and the plateau of the dependence is shifted at a higher frequency of the electric field. However, the frequency of relaxation almost does not depend on the alginate sample (ca. 6 kHz).

According to the presented results, there is a correlation between the registered effect from the particles covered by alginate and the fraction of guluronic residues. The electro-optical effect increases with the increase in the moiety of the G-residues (or decrease in the M/G ratio). It was supposed that the correlation results from the stronger interaction between the Ca^2+^ and alginate molecules, whereby the divalent cations have a contribution to the creation of the effect.

#### Alginate/Chitosan Film

To investigate the influence of the alginate properties on the formation of stable polysaccharide film, in the present study, an alginate/chitosan bilayer was produced on the model oxide particles. 

**Figure 6:**
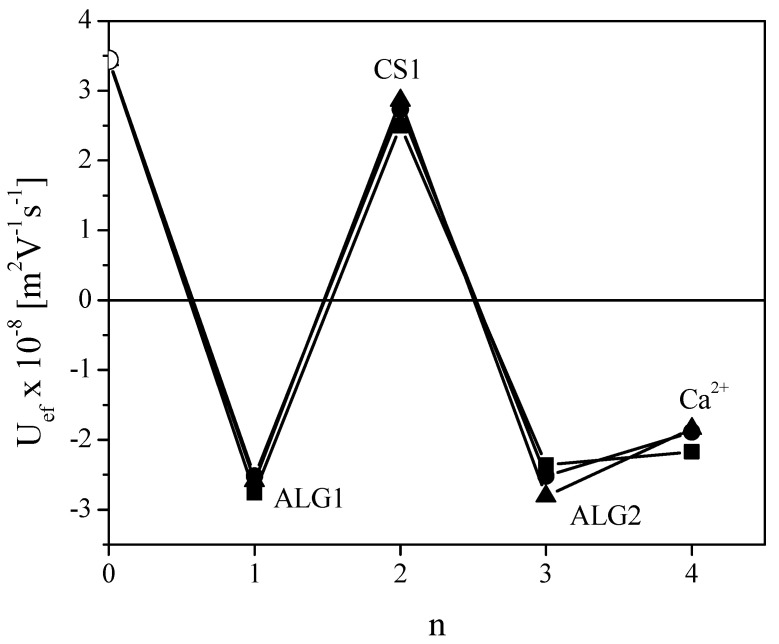
Dependence of the electrophoretic mobility, U_ef_, of β-FeOOH particles as a function of a number of adsorbed steps of chitosan/alginate film formed from A082 (▲), A122 (■), and A047 (●). Open symbol corresponding to the mobility of bare β-FeOOH particles first alginate layer (ALG1) chitosan layer (CS1) and second alginate layer (ALG2).

Figure 6 presents the variation in U_ef_ of the polymer-coated particles as a function of the number of adsorption steps. The experimental results indicate the achievement of an overcharging of the particle charge after the adsorption of alginate or chitosan. Adding CaCl_2_ to the suspension after deposition of the second alginate layer leads to a slight decrease in the U_ef_ because of a partial screening of the charge in the last deposited layer.

**Figure 7:**
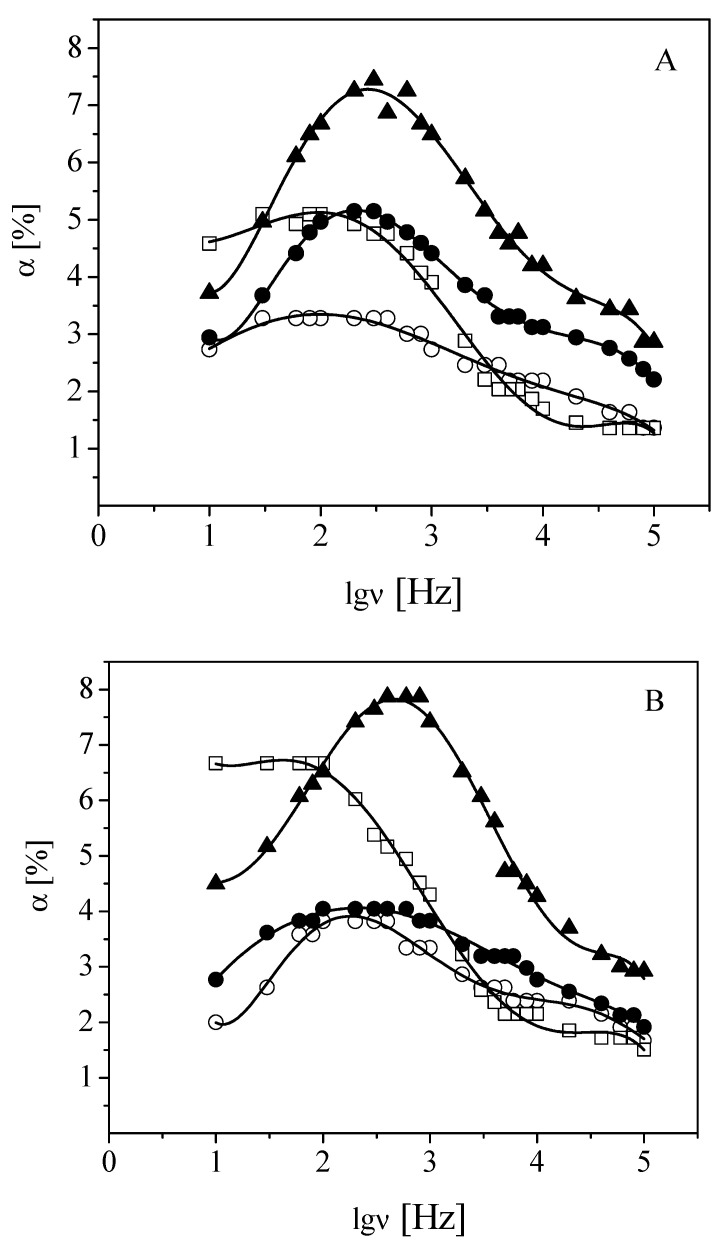

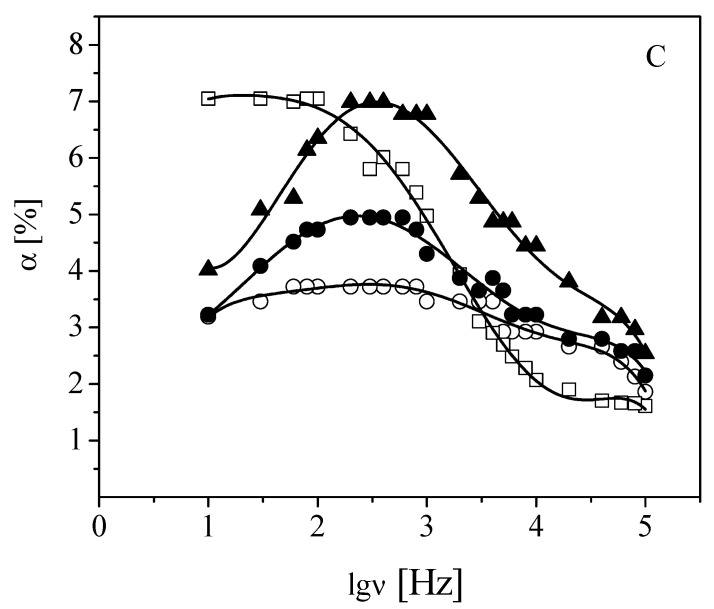
Frequency dependence of the electro-optical effect, α, from a stabilized suspension of particles with adsorbed ALG1 (○), CS1 (□), ALG2 (●), and in the presence of Ca^2+^ (▲) for a film formed from alginate A082 (**A**), A122 (**B**), and A047 (**C**). The concentration of polysaccharides is 10^−2^ mg/mL. The electric field strength is 2.3 × 10^4^ V/m.

Figure 7 presents the registered electro-optical behavior of suspension of particles covered with alginate/chitosan film at a low energy of orientation. As can be seen, the subsequent adsorption of alginate and chitosan or the addition of Ca^2+^ results in the different characteristic shapes of the frequency dependence. According to the presented results in the previous section, we supposed that the polarization of condensed Na^+^ counterions along the polyion contour is responsible for the observed electro-optical response from a suspension of particles with the last adsorbed layer from the alginate. Moreover, the estimated ν_cr, exp_ for the particles covered by alginates with different physicochemical characteristics might result from the polarization of counterions at specific distances according to the molecular structure (G-, M-, or MG-blocks). That is why the ν_cr, exp_ does not depend on the number of the adsorbed alginate layer formed from different polymer samples. The adsorption of the second alginate layer leads to an increasing registered effect from the suspensions increasing because of the increase in the number of polyelectrolyte molecules (or charges) on the increasing particle surface.

In the presence of Ca^2+^ ions, the frequency of the effect is ca. 6 kHz and does not depend on the physicochemical characteristics of the alginate (Figure 7). Therefore, it is expected that a similar polarization process is responsible for the observed electro-optical behavior from a suspension of the particles stabilized by the alginates. Three possible reasons exist to explain the experimental results: (i) a decrease in the mobility of the Na^+^ ions because of the variation in the conformation of the adsorbed chains in the presence of CaCl_2_, (ii) a decrease in the thickness of the electric double layer of the particles because of the increase in the ionic strength of suspension, or (iii) the participation of the Ca^2+^ ions in the polarization process.

To analyze the registered electro-optic response, we must take into consideration the ion-binding ability of the alginates. The ion affinity of the alginates originally allocated to the specific interactions predominantly between the G-residues and divalent cations that result in the formation of junctions. According to the “egg-box” model proposed by [13,14,15], each cross-linking cation interacts with two adjacent G-residues along the polymer chain as well as with two G-residues of another chain, through the formation of the junction zone. It has been shown that 4:1 is a suitable ratio between the G-residues and cross-linking Ca^2+^ cations for successful ion binding [23,24].

In the present study, the ratio between the G-residues (at 10^−2^ mg/mL alginate) and the concentration of Ca^2+^ cations (at 2.5 × 10^−5^ M CaCl_2_) is ca. 1:1 for all polymer samples. However, the sequence of G and M residues, as well as the length of G-blocks are unknown. Therefore, it is not possible to conclude if the concentration of cross-linking cations is high enough to ensure the formation of the ion hydrogel structure on the surface of the particle.

**Figure 8:**
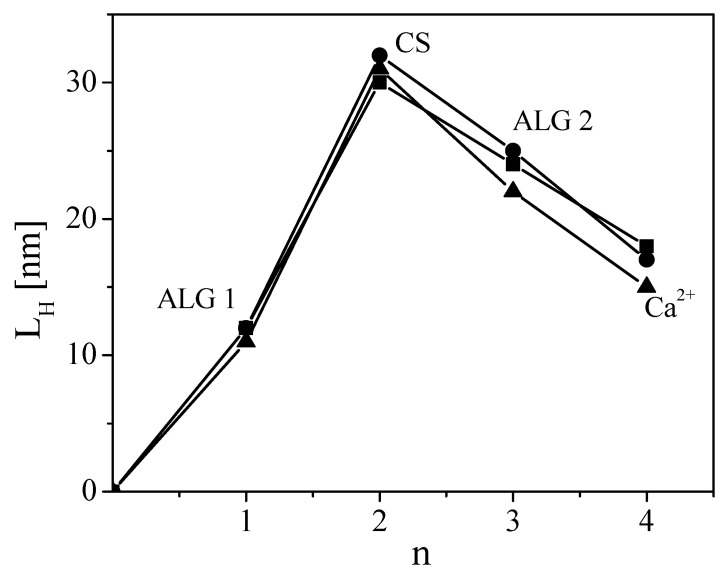
Dependence of the hydrodynamic thickness of particles with chitosan/alginate film formed from ALG-A (▲), ALG-B (■), and ALG-C (●) as a function of the adsorbed steps.

The decrease in the thickness of the polymer layer after adding Ca^2+^ ions is an indicator of the variation in the film structure (Figure 8). According to Equation 8, the decrease in the length of polarization of the Na^+^ ions will correlate with the increase in the frequency of relaxation of the electro-optical effect, but this is not observed for all alginate samples (ALG-C). Consequently, the assumption for the effect of decreasing the mobility of the Na^+^ ions cannot be interpreted as synonymous.

The participation of the Ca^2+^ ions in the polarization process could be another reason for the observed electro-optic response from the suspension of particles with adsorbed alginate.

In the framework of the “egg-box” model, “in view of the strong binding of divalent cations in the junctions, the term “chemical bonding” seems appropriate to describe the interactions between the cross-linking ion and the uronic moieties”. The “chemically bonded” ions can be considered as “localized” in the specific site on the chain for a residence time much longer than the “territorially” condensed counterions and the time for exchange of the condensed ions with the free ions in the solution [35].

Van den Hoop et al. [19,22] also supposed that the association of divalent counterions to the polyion is due to the formation of chemical bonds rather than through condensation since the binding occurs at a polyelectrolyte charge density below the critical value. Moreover, Siew and Williams [17] estimated the charge parameters for two alginate samples with different M/G ratios and confirmed the suggestion of the association of divalent ions to the carboxylate groups along the polyion chains through chemical bonding. The calculated values for the charge spacing and the fraction of condensed or free monovalent counterions are very close to those presented in our study (Table 1).

It Is important to mention that, in the framework of the original concept of condensation theory, the short-range and site-specific bonding interactions are not considered. The condensed counterions are loosely associated with the polyelectrolyte chain and no specific affinity modifies the expected total fraction of the condensed ions. Hence, only the fluctuations of counterions within the condensation volume near the polyion chain and the exchange of condensed counterions with free counterions from the solution are allowed.

The description of the problem for the application of the condensation theory for the system with a mixture of counterions with different valences is already considered. Within the different theoretical approaches and mathematical methods, the strong site-specific bonding of divalent cations responsible for the ion-induced chain interactions of alginates is a well-described example.

Paoletti et al. [36,37] have developed an extension of the condensation theory for a system of linear polyelectrolytes in a mixture of mono- and divalent counterions. The results predicted via the model indicated the cooperative binding of divalent counterions (even at very low concentrations) in a solution containing high concentrations of monovalent ions and a low charge density of polyelectrolyte.

According to Manning [18], in the presence of monovalent and divalent counterions, ion binding would depend on the value of the dimensionless ξ parameter. When ξ > 1, the divalent ions will bind to the polyion. In the case that all the divalent ions in the solution are bound and 0.5 < ξ_effective_ < 1, there is no binding of monovalent counterions. However, if ξ_effective_ > 1, the monovalent ions will bind until ξ_effective_ reduces to 1. Taking into account the theoretical considerations for mixed-valence polyelectrolyte systems, in the present study, it is assumed that the variation in the electro-optical behavior of the particles with adsorbed alginate molecules is affected by the presence of divalent ions in the polymer layer. Since the territorial condensed counterions and site-specific bonding interactions usually occur simultaneously, it is difficult to experimentally identify the contribution of their fractions. The electro-optics methods can distinguish the contribution of the polarization of the diffuse ions from the double electric layer of the particles from the polarization of the counterions with lower mobility, but, in the present study, it is complicated to separate the contribution of the monovalent condensed counterions and divalent cross-linking counterions (or chemically bounded) with lower mobility compared to the diffuse ones. The present study of the similar electro-optical behaviors from the suspensions of particles covered with alginates with different physicochemical characteristics supposed that there was a contribution of a similar polarization process of counterions from the polyelectrolyte adsorbed on the surface.

According to Equation 2, from the experimental value for the frequency of relaxation, it can be estimated that the polarization length for the Na^+^ (ca. 72 nm) and Ca^2+^ (ca. 55 nm) counterions ($D_{{Na}^{+}}^{C}$ = 9.8 × 10^−7^ cm^2^ s^−1^ and $D_{{Na}^{+}}^{C}$ = 5.8 × 10^−7^ cm^2^ s^−1^).

The dependencies from the particles with adsorbed chitosan layers are presented in Figure 8. These indicate there is a plateau at a lower frequency of the electric field (below 1 kHz). The experimental value of ν_cr, exp_ is ca. 4 kHz and does not depend on the alginate sample. In correspondence with our previous studies, it can be supposed that the electro-optical behavior of the particles covered by a chitosan layer is defined by the polarization of condensed counterions along the polyion chain deposited in the last adsorbed step [28].

## 3. Conclusions

The comparative investigation of the effect of the physicochemical characteristics of alginate on the electrical properties and stability of dispersion of polymer-coated colloid particles was performed by using electrokinetic and electro-optic methods.

The electrokinetic response from the suspension of particles in the presence of different concentrations of alginate or CaCl_2_ has not indicated that there is a dependence on the alginate characteristics. On the other side, the analysis of the electro-optical behaviour and dynamics of counterion charge near the vicinity of adsorbed polyion is shown that the experimental values of the frequency of relaxation of the electro-optical effect are higher compared to the theoretical ones. Therefore, it was supposed that polarization of condensed Na^+^ counterions becomes on the smaller distance along the polyion contour (G-, M- or MG-blocks). Since the residue sequences of the alginate molecules in samples are unknown, the estimated ν_cr, exp_ might result from the polarization of counterions at specific distances according to the molecular structure (G-, M- or MG-blocks).

In the presence of Ca^2+^ was registered that the electro-optical behaviour of the particles with adsorbed alginate almost does not depend on the polymer characteristics, but it is affected by the presence of divalent ions in the polymer layer. The electro-optical effect increases as the moiety of the G-residues increases (or the M/G ratio decreases) but the relaxation frequency of the effect does not depend on the alginate sample.

## 4. Materials and Methods

### 4.1. Materials

**Scheme 1:**
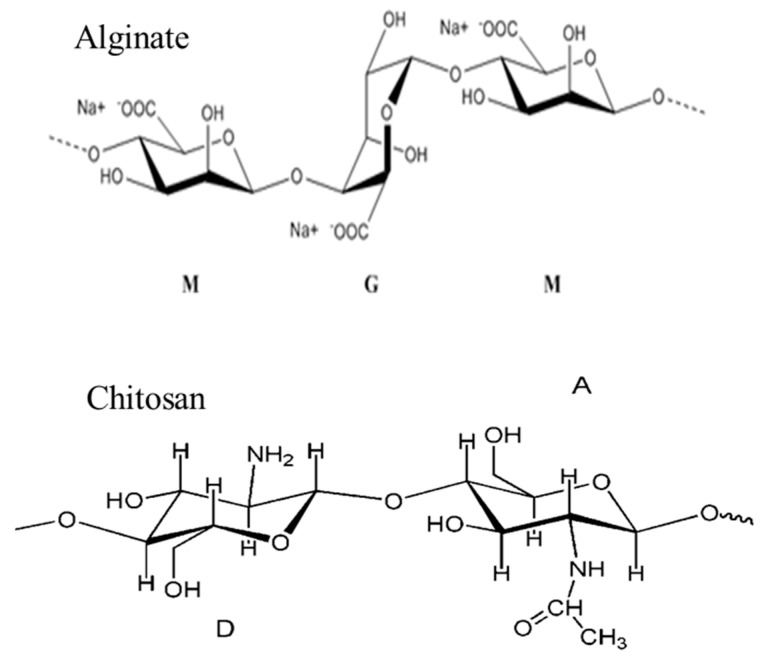
Schematic chemical structure of alginate sodium salt (guluronate, G, and mannuronate, M, residues) and chitosan (acetylated, A, and deacetylated, D, monomers).

The alginates, sodium salt (ALG) were obtained from FMC Technologies (Kongsberg, Norway) The characteristics of the used samples provided by the manufacture are presented in Table 2. The chitosan (CS) (degree of deacetylation 75–85%, medium molecular weight 190–310 kDa) was purchased from Sigma–Aldrich (Saint Louis, MO, USA). The polyelectrolyte solutions were prepared with a concentration of 1 mg/mL. For the preparation of the stock chitosan solution, the sample was dissolved in a solution of hydrochloric acid pH 4.0, whereas the alginate sample was dissolved in double distilled water pH 5.08. The chemical structures of the polysaccharides are presented in Scheme 1.

**Figure 9:**
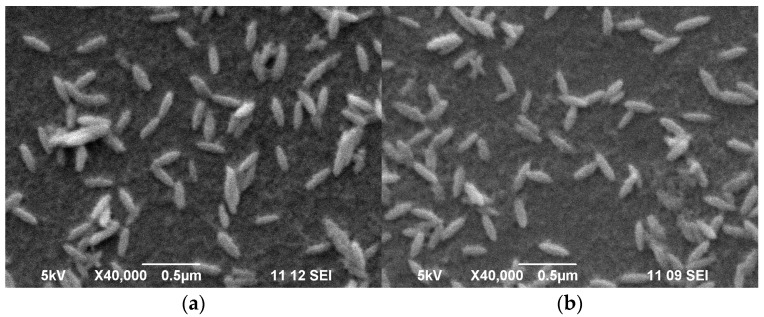
Representative SEM photographs of bare β-FeOOH particles (**a**) and particles with adsorbed alginate/chitosan film (**b**).

The polysaccharides were adsorbed on the model ellipsoidal β-FeOOH particles (Figure 9), which were synthesized following a well-established experimental procedure [40].

### 4.2. Methods

#### 4.2.1. Electro-Optic Method

The electric light scattering method was used to characterize the variation in the electrical and geometrical properties of the anisometric model particles upon the applied electric field. Detailed information about the method and the electro-optical experiment were presented in previous papers [26,27,28,29].

The electro-optical effect is defined as the difference between the intensities of the scattered light from suspension in the presence and absence of an electric field [41].

(3) $$\alpha =\frac{I_{E}-I_{0}}{I_{0}}$$

The transient process of the particle disorientation after switching the electric field off permits estimating the rotation diffusion coefficient, *D_r_*, relative to the particle size. For the prolate ellipsoid, *D_r_* is provided by:

(4) $$D_{r}=\frac{kTp^{2}}{4\eta \upsilon \left(p^{4}-1\right)}\left[-1+\frac{2p^{2}-1}{2p\sqrt{p^{2}-1}}ln\frac{p+\sqrt{p^{2}-1}}{p-\sqrt{p^{2}-1}}\right]$$

where *p* is the axial ratio *a*/*b*, *η* the viscosity of the suspending medium, and *v* is the particle volume [42]. The hydrodynamic thickness of the adsorbed layer, L_H_, was estimated from the change in the particle dimensions after polyelectrolyte adsorption.

#### 4.2.2. Microelectrophoresis

The electrophoretic mobility, U_ef_, was evaluated using Mark II apparatus (Rank Brothers Ltd, Cambridge, UK).

#### 4.2.3. Scanning Electron Microscopy (SEM)

The particles were visualized via scanning electron microscopy. The images of the bare particles and particles covered with alginate/chitosan film were captured using a scanning electron microscope JEOL JSM 6390 (JOEL USA Inc., Peabody, MA, USA).

#### 4.2.4. Preparation of the Suspensions

The samples for the stability experiments were prepared by adding the suspension of β-FeOOH (particle concentration 8 × 10^−3^ mg/mL, pH 4 adjusted with HCl) to the alginate solution with a concentration of 1 mg/mL to achieve the final concentration of the alginate in suspension in the range of from 10^−5^ to 4 × 10^−2^ mg/mL and stirring for 20 min at room temperature (ca. 24 °C). Every sample (total volume of 10 mL) was prepared separately from the stock suspension of particles. The concentration of CaCl_2_, added only to the stabilized suspensions (10^−2^ mg/mL alginate) varied from 1 × 10^−5^ to 7.5 × 10^−4^ M.

To form the ALG/CS bilayer, the suspension (10 mL) of the ALG-coated particles (10^−2^ mg/mL alginate) was added to the solution (0.1 mL) of positively charged chitosan (final concentration in solution is 10^−2^ mg/mL). The free polysaccharide molecules in the dispersion were removed via centrifugation (at 4500 rpm for 20 min). The settled particles were re-dispersed via sonication in an acidic solution with pH 4. After the deposition of the second alginate layer, a low concentration of CaCl_2_ (2.5 × 10^−5^ M) was added to the suspension.

## Figures and Tables

**Table 1:** Estimation of the fraction of released free counterions upon adsorption of alginate polyion (in absence of CaCl_2_) in the presence of condensed counterions, polyion contour length (L), average monomer length (b), Manning’s parameter (ξ), total fraction of free (β = 1/ξ) counterions, experimental and theoretical values of frequency of relaxation of the effect (ν_cr,exp_ and ν_cr, theor_ calculated according to Equation (2)), length of polarization of counterions calculated from ν_cr, exp_ (L*, calculated according to Equation (2)), and number of glucuronic residues (estimated from M_w_ and M/G ratio of the molecules).

Alginate Sample	L [nm]	b [nm]	ξ	β_Manning_	ν_cr,exp_ [kHz]	ν_cr, theor_, [kHz]	L* [nm]	G_residues_
A082	1643	0.47	1.51	0.66	5	0.046	158	1912
A122	1070	0.48	1.49	0.67	3	0.042	204	1604
A047	959	0.37	1.92	0.52	17	0.136	86	1414

**Table 2:** Characteristics of alginate samples: M_n_ (number average molecular weight), M_w_ (weight average molecular weight), M_z_ (higher average molecular weight), polydispersity index (M_w_/M_n_), and M/G ratio (the manufacturer provides the characteristics).

Alginate Sample	A082	A122	A047
M_n_, kDa	341.70	309.90	227.40
M_w_, kDa	671.80	687.30	496.70
M_z_, kDa	958.30	1081.40	705.00
Polydispersity index	1.97	2.22	2.18
M/G ratio	0.82	1.22	0.47

## Data Availability

Not applicable.
